# Cytokinins Are Age- and Injury-Responsive Molecules That Regulate Skeletal Myogenesis

**DOI:** 10.3390/ijms262010136

**Published:** 2025-10-18

**Authors:** Farnoush Kabiri, Zeynab Azimychetabi, Dev Seneviratne, Lorna N. Phan, Hannah M. Kavanagh, Hannah C. Smith, R. J. Neil Emery, Craig R. Brunetti, Janet Yee, Stephanie W. Tobin

**Affiliations:** 1 Environmental and Life Sciences Graduate Program, Trent University, Peterborough, ON K9L 0G2, Canada; 2 Department of Biology, Trent University, Peterborough, ON K9L 1Z8, Canada

**Keywords:** skeletal myogenesis, cytokinins (CTKs), isopentenyladenine (iP), isopentenyladenosine (iPR), kinetin, i^6^A

## Abstract

Myogenesis is a tightly regulated process essential for embryonic development, postnatal growth, and muscle regeneration. We recently identified that cytokinins (CTKs), a class of adenine-derived signaling molecules originally characterized in plants, are present in cultured skeletal muscle cells. The most abundant type of cytokinins detected within cultured muscle cells was isopentenyladenine (iP) in its nucleotide, riboside, and free base derivatives. The purpose of this study was to determine whether CTKs are also present in regenerating muscle tissue in vivo and to characterize the effects of iP and its riboside form, isopentenyladenosine (iPR), on muscle cell proliferation and differentiation. These effects were observed relative to adenine and adenosine, and to a second class of cytokinins with a large aromatic side chain, kinetin (the free base), and kinetin riboside. Cardiotoxin was used to induce muscle injury and repair processes in the gastrocnemius of 3- and 12-month-old mice. Samples were collected 3- and 7 days post-injury for ultra high-performance liquid chromatography tandem mass spectrometry with electrospray ionization (UHPLC-(ESI+)-HRMS/MS). Four CTKs (N^6^-benzyladenine (BA), dihydrozeatin-9-N-glucoside (DZ9G), isopentenyladenosine (iPR), and 2-methylthio-isopentenyladenosine (2-MeSiPR) were detected. 2-MeSiPR levels were significantly influenced by aging, as this CTK was increased in response to injury only in the younger mice. Treatment of C2C12 myoblasts with 10 µM of isopentenyladenosine (iPR) or kinetin riboside reduced cell proliferation, whereas iP (the free base) increased proliferation in a biphasic response. During differentiation, both iPR and kinetin riboside impaired myotube formation, while the free-base forms of iP and kinetin had no effect. Our data establishes that CTKs are present within muscle tissue and highly responsive to injury and aging. Furthermore, the biological activities of CTKs in muscle cells are influenced by structural modifications, including riboside conjugation and side chain composition. Understanding these differences provides insight into the distinct roles of CTKs in muscle cell metabolism and differentiation, offering potential implications for the use of exogenous CTKs in muscle biology and regenerative medicine.

## 1. Introduction

Skeletal muscle development and regeneration are critical biological processes essential for movement, metabolism, and overall health. Myogenesis, the formation of skeletal muscle, is a highly regulated process involving the proliferation, differentiation, and fusion of myogenic precursor cells known as myoblasts into multinucleated myotubes and mature myofibers [1]. This process is orchestrated by myogenic regulatory factors (MRFs), including MyoD, Myf5, and myogenin (Myog), which control the progression from proliferating satellite cells and committed myoblasts to fully differentiated muscle fibers [2]. The expression of muscle-specific proteins, such as myosin heavy chain (MyHC) and muscle creatine kinase (Mck), serves as a hallmark of myotube maturation. Given that skeletal muscle accounts for approximately 40% of total body mass and plays a crucial role in metabolic homeostasis, disruptions in myogenesis have been implicated in numerous pathological conditions, including muscular dystrophies, sarcopenia, and cachexia, thereby underscoring the need for therapeutic strategies targeting muscle regeneration [3].

Cytokinins (CTKs) are a class of adenine-derived signaling molecules that regulate cell proliferation and differentiation across various biological systems. Although primarily recognized for their roles in plant development, CTKs have been increasingly studied for their biological relevance in animal and human physiology [4]. These molecules exist in different structural forms, such as free bases, ribosides, nucleotides, and other conjugates, each exhibiting distinct biological activities and functions [5]. Free-base CTKs are generally the most biologically active in plants, whereas nucleotide and riboside forms function as biosynthetic intermediates or signaling molecules with varying degrees of activity [6]. The metabolic regulation of CTKs in mammalian cells remains an area of emerging interest, with accumulating evidence suggesting that these molecules influence cellular processes beyond the plant kingdom.

Two primary pathways govern CTK biosynthesis: the de novo adenylate pathway and the tRNA degradation pathway [5]. In plants, the adenylate pathway produces isoprenoid CTKs such as isopentenyladenine (iP), trans-zeatin (tZ), and dihydrozeatin (DZ), while the tRNA degradation pathway generates iP, cis-zeatin (cZ), and 2MeS CTKs. In contrast, mammalian CTK biosynthesis relies solely on the tRNA degradation pathway mediated by isopentenyl transferase (TRIT1), an enzyme that modifies A37 in a subset of tRNAs [7,8]. Upon degradation of prenylated tRNA, isopentenyl adenosine monophosphate (iPMP) is released, acting as a precursor for subsequent CTK metabolism, such as conversion to the riboside, isopentenyl adenosine (iPR aka i^6^A). Despite the structural similarities between plant and mammalian CTKs, the functional implications of these molecules in animal systems remain poorly understood, necessitating further investigation.

The therapeutic benefit of exogenous CTKs has been evaluated in various cell and animal systems demonstrating that CTKs have neuroprotective, immunomodulatory, and antiproliferative effects that have potential therapeutic applications in human health [9,10,11,12,13,14]. Furthermore, CTK expression has been identified in mammalian tissues [15,16], including canine skeletal muscle [17], indicating a possible physiological role in muscle development and regeneration. The ability of muscle cells to both produce and respond to CTKs implies that these molecules may influence myogenesis and muscle repair mechanisms. However, as endogenous CTKs are difficult to measure, the precise molecular mechanisms underlying CTK function in skeletal muscle remain unexplored.

To address this knowledge gap, we collected injured muscle tissue from young (3-month-old) and old (12-month-old) mice at both 3- and 7 days post-injury and screened for endogenous cytokinins using mass spectrometry. We also investigated the effects of treatment with two types of exogenous cytokinins on myogenesis in vitro: the iP type characterized by an isoprenyl chain, which we previously detected in cultured myoblasts [18], and kinetin, an aromatic form derived from DNA degradation. The two types of CTKs were chosen for their reported roles in other mammalians systems. The iP types have been suggested to act as tumor suppressors and reduce proliferation while the kinetin types have been shown to have antioxidant and DNA repair potential and act as a neuroprotectant [4]. Both the free base and riboside forms of these cytokinins, as well as adenine and adenosine, were added to cultured myoblasts to determine the effects of these similar molecules on muscle cell proliferation and differentiation.

## 2. Results

### 2.1. Cytokinins Are Present in Mouse Skeletal Muscle Tissue and Demonstrate Age- and Injury-Dependent Profiles

**Figure 1:**
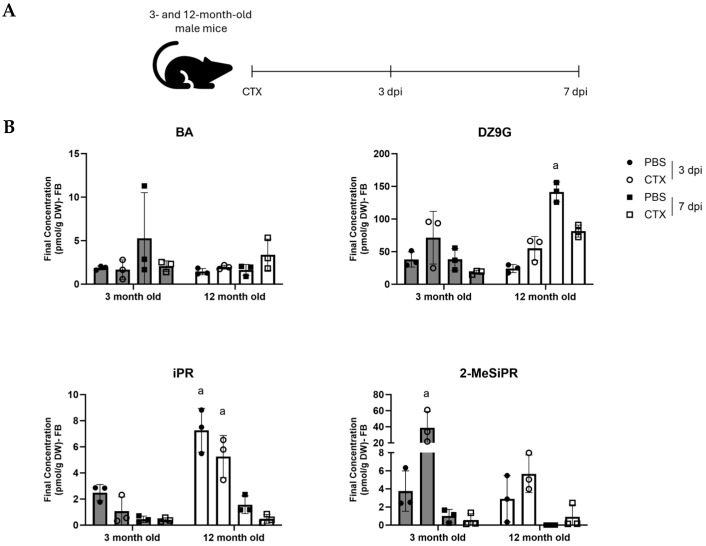
Endogenous cytokinin levels in the injured muscle of young and old mice. (**A**) Muscle injury timeline. The gastrocnemius of male mice (aged 3 or 12 months) was injured using cardiotoxin (CTX) and isolated for mass spectrometry either 3- or 7 days post injury (dpi). The contralateral control leg received equal volume injections of phosphate-buffered saline (PBS). (**B**) Cytokinin levels in mouse skeletal muscle. Samples were normalized to gram dry weight (g DW) of the muscle tissue. Data are shown as mean ± SD (n = 3). A two-way ANOVA test with Sidak’s correction (*p* < 0.05) was used to determine statistical significance where a = *p* < 0.05 compared to all other treatments. N^6^-benzyladenine (BA), dihydrozeatin-9-N-glucoside (DZ9G), isopentenyladenosine (iPR), and 2-methylthio-isopentenyladenosine (2-MeSiPR).

Our team has previously shown that canine muscle tissue and cultured myoblasts produce cytokinins [17,18]. To better understand the regulation and potential role of endogenous cytokinins, we profiled cytokinins in injured muscle tissue from young and old male mice (3- and 12 months old) at both 3- and 7 days post-injury (dpi; Figure 1A). These timepoints were selected as skeletal muscle injury in young mice typically develops a strong inflammatory response 3 dpi that is resolved after 7 days, however older mice have a delayed response [19]. The contralateral leg from each mouse received injections of saline and were also used in the analysis.

Of the 28 cytokinins probed [20], 4 were detected: N^6^-benzyladenine (BA), dihydrozeatin-9-N-glucoside (DZ9G), isopentenyladenosine (iPR), and 2-methylthio-isopentenyladenosine (2-MeSiPR) (Figure 1B). In general, BA AMD DZ9G were the least and most abundant CTKs detected across the 8 treatment groups, respectively. Derivatives of iP, which we had previously detected in cultured muscle cells [18], were also present in muscle tissue and tended to show age- or injury-responsive profiles. For example, iPR levels were signifcantly higher in the 12 month old muscles at 3 dpi in both the CTX injured legs and the contralateral control muscles. In contrast, 2-MeSiPR was significantly increased in the 3 month old CTX-injured muscle at 3 dpi compared to other groups. 2-MeSiPR was not detected in the 12 month old muscle saline-injected leg at 7 dpi.

### 2.2. Cytokinins Have Concentration- and Side Chain-Dependent Effects on C2C12 Myoblast Proliferation and Viability

**Figure 2:**
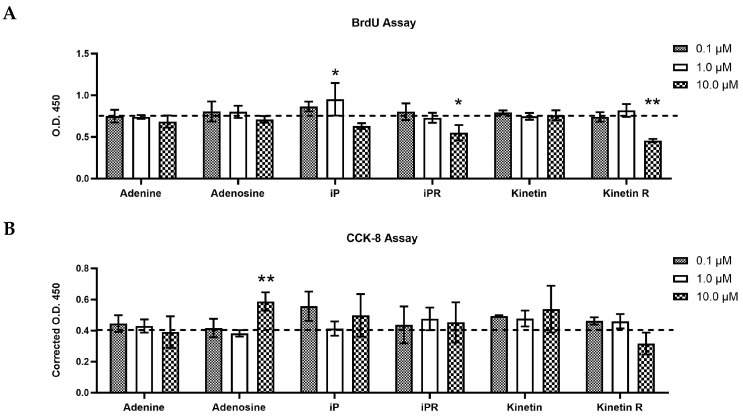
Effect of cytokinins on the proliferation (**A**) and viability (**B**) of C2C12 myoblasts. (**A**) The effect of cytokinins on BrdU incorporation in C2C12 myoblasts in serum-free media. (**B**) Cell viability of C2C12 myoblasts in serum-free media as measured via the CCK-8 assay. In both 2A,B, CTK treated cells were compared to a DMSO control, as indicated by the horizontal dashed line in the graphs, and cells were treated with CTKs for a total of 24 h. Data are shown as mean ± SD (n = 4). A one-way ANOVA test with Dunnett’s correction (*p* < 0.05) was used to determine statistical significance relative to the DMSO control where * *p* < 0.05, ** *p* < 0.01. iP = isopentenyladenine; iPR = isopentenyladenosine; Kinetin R = Kinetin riboside; O.D. = Optical density.

Given that we have now detected iP types in vitro [18] and in vivo (present study) we tested the effect of exogenous iP types on myogenesis. For comparison we included an aromatic CTK, kinetin, which has not been detected endogenously but has been suggested to have beneficial cellular effects. To begin, the impact of isopentenyladenine (iP), kinetin, and their riboside derivatives on the proliferation and viability of C2C12 myoblasts was evaluated using BrdU incorporation and CCK-8, respectively (Figure 2). In both experiments, myogenic C2C12 cells were exposed to concentrations of 0.1 µM, 1.0 µM, and 10 µM of each compound for a duration of 24 h in serum-free media. These concentrations were selected based on previous studies wherein L6 or C2C12 myoblasts were treated with exogenous CTKs in similar ranges [21,22]. Considering that cytokinins (CTKs) are adenine-derived compounds, the effect of adding adenine and adenosine were also examined.

Treatment with 10 µM of isopentenyladenosine (iPR) and kinetin riboside significantly decreased BrdU incorporation, indicating reduced cell proliferation (Figure 2A). In contrast, 0.1–1.0 µM of iPR and kinetin riboside had no effect. Treatment with isopentenyladenine (iP) elicited a biphasic effect, where 1 µM significantly increased BrdU incorporation, but 0.1 µM and 10 µM had no effect. Adenine, adenosine, and kinetin did not affect cell proliferation. Based on the CCK-8 assay, cell viability of myoblasts was not affected by cytokinins, though treatment with adenosine (10 µM) significantly enhanced myoblast viability (Figure 2B).

### 2.3. Effects of iP, Kinetin, and Their Riboside Forms on Myoblast Migration and Wound Healing

**Figure 3:**
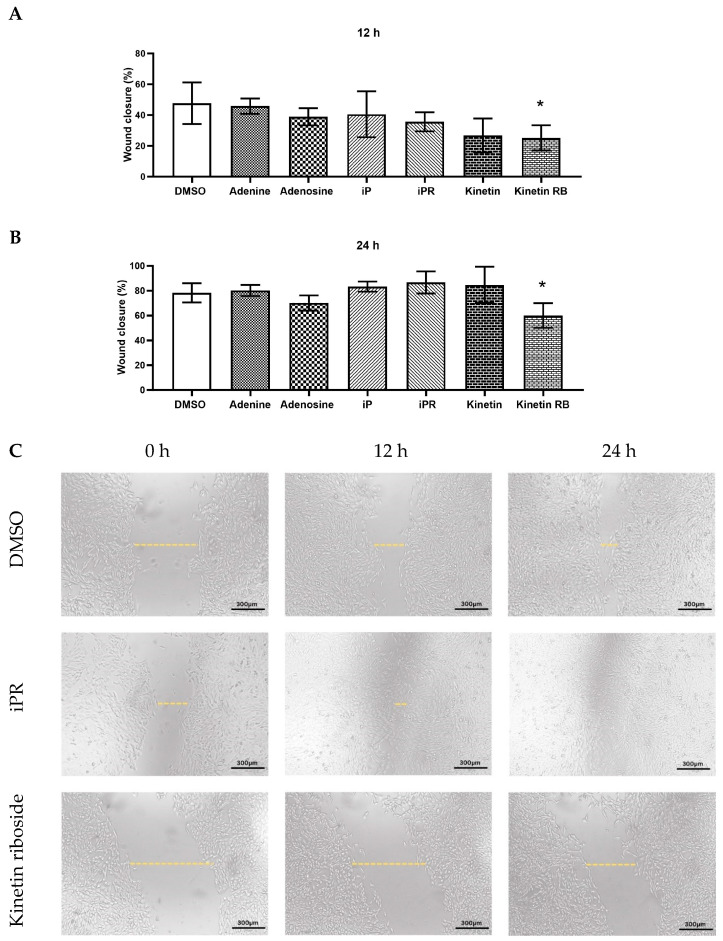
The effect of cytokinins on wound repair of C2C12 myoblasts under normal growth conditions. Wound closure was assessed at 12 h (**A**) and 24 h (**B**) post-treatment with the indicated treatment (10 µM) and compared to the DMSO-treated control group. (**C**) Representative images of the scratch wound showing wound closure over a 24 h period. Data are shown as mean ± SD, n = 3–4. A one-way ANOVA test with Dunnett’s correction (* *p* < 0.05) was used to determine statistical significance relative to the DMSO control. The size of the scratch is indicated by a dashed yellow line. iP = isopentenyladenine; iPR = isopentenyladenosine; Kinetin R = Kinetin riboside.

A scratch assay was conducted to evaluate the effects of CTKs on myoblast migration and wound healing. C2C12 cells were grown to confluency in complete growth medium. A scratch was made to the layer of cells supplemented with 10 mM of each treatment and the migration of cells back into this area was evaluated at 12 h and 24 h post-injury. The concentration of 10 µM was chosen for these experiments as lower concentrations had no effect (aside from iP at 1 µM) on proliferation or viability (Figure 2). We observed a marked reduction in wound healing capacity in cells treated with kinetin riboside (10 µM) compared to the control group at both 12 h (Figure 3A) and 24 h post-treatment (Figure 3B). These findings align with the repressive effects of kinetin on cell proliferation as assessed by the BrdU assay (Figure 2A).

### 2.4. Isopentenyladenosine (iPR) and Kinetin Riboside Reduce Myotube Viability and Differentiation of C2C12 Cells

**Figure 4:**
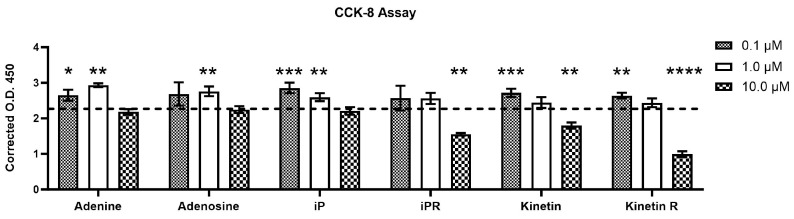
Cell viability of differentiating C2C12 cells when co-cultured with cytokinins. Viability of C2C12 cells undergoing differentiation with varying concentrations of cytokinins in differentiation media (DM) was evaluated using the CCK-8 assay after 144 h in differentiation medium. The DMSO control group, illustrated by the horizontal dashed line in the graph, serves as the reference for comparison. Data are shown as mean ± SD, n = 4. A one-way ANOVA test with Dunnett’s correction (*p* < 0.05) was used to determine statistical significance relative to the DMSO control, where * *p* < 0.05, ** *p* < 0.01, *** *p* < 0.001, and **** *p* < 0.0001. iP = isopentenyladenine; iPR = isopentenyladenosine; Kinetin R = Kinetin riboside. O.D. = Optical density.

To assess the effects of CTKs, adenine, and adenosine on cell viability during later stages of differentiation, C2C12 cells were maintained in differentiation medium (DM) and treated with 0.1, 1.0, or 10.0 µM of each compound for 144 h prior to CCK-8 viability assessment. At lower concentrations of 0.1–1.0 µM, adenine and adenosine tended to increase cell viability (Figure 4). This effect was similarly observed in response to isopentenyladenine, kinetin, and kinetin riboside, which increased viability at lower concentrations, whereas iPR did not. At 10 µM, iPR, kinetin, and kinetin riboside significantly reduced cell viability (Figure 4). These findings indicate that kinetin riboside and isopentenyladenosine exert inhibitory effects on the viability of differentiating myoblasts in a dose-dependent manner and that these effects differ from non-modified adenine and adenosine.

**Figure 5:**
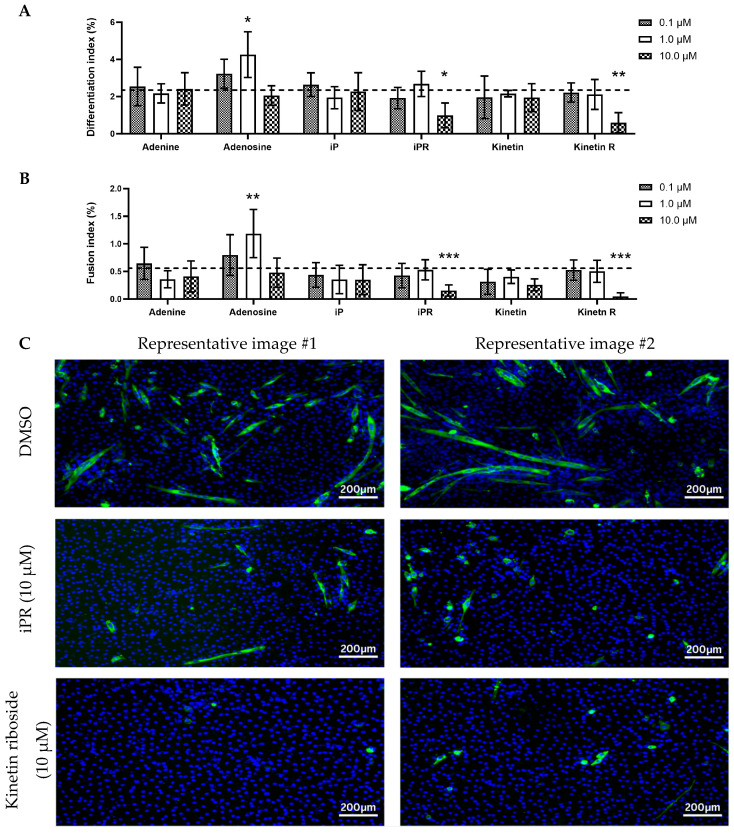
The effect of cytokinins on skeletal myogenesis. (**A**) Differentiation and (**B**) fusion indices after 144 h of cultivation in differentiation media. The differentiation index, representing the proportion of cells that differentiated into myotubes, was calculated as the percentage of myosin heavy chain-positive cells. The fusion index is represented as the percentage of multinucleated myosin heavy chain-positive cells. Differentiation and fusion indices of treated cultures were compared with the control group, represented by the horizontal dashed line (n = 6). Data are shown as mean ± SD. A one-way ANOVA test with Dunnett’s correction (*p* < 0.05) was used to determine statistical significance where * *p* < 0.05, ** *p* < 0.01, and *** *p* < 0.001. (**C**) Representative immunofluorescent staining images of C2C12 cells treated with 10 µM of iPR or kinetin riboside compared to the DMSO control. Cells were stained for myosin heavy chain (green) to assess myogenic differentiation, and nuclei were counterstained with DAPI (blue). Two representative images per treatment are shown. iP = isopentenyladenine; iPR = isopentenyladenosine; Kinetin R = Kinetin riboside.

To further evaluate the impact of CTKs on differentiation, myogenic indices were analyzed through immunostaining for myosin heavy chain (MyHC), a late-stage differentiation marker. The differentiation index, which is the proportion of cells that differentiated into myotubes, revealed that treatment with 10 µM of either iPR or kinetin riboside inhibited myogenesis (Figure 5A). Similarly, kinetin riboside and iPR significantly reduced the number of multinucleated cells as measured by the fusion index (Figure 5B). A biphasic effect of adenosine on myogenesis was observed, wherein 1.0 µM of adenosine increased both differentiation and fusion indices but this was not observed at the higher and lower concentrations of adenosine.

## 3. Discussion

Originally discovered and best characterized in plants, our understanding of CTKs now extends to mammalian systems, with recent analyses revealing their potential functions in regenerative medicine, including skeletal muscle differentiation [18,22]. We profiled CTK levels in regenerating muscles of young and old mice and investigated the role of isopentenyladenine (iP) and kinetin type CTKs on myogenesis. We found that CTKs have injury- and age-dependent profiles in vivo and that exogenous treatment with CTKs affect muscle cell proliferation and differentiation but that these effects depend on cytokinin concentration, side chain modification, and whether the cytokinin is a free base or riboside. We observed that the riboside forms of both isopentenyl and kinetin repress cell proliferation and differentiation while iP (the free base) increases cell proliferation in a biphasic manner.

### 3.1. CTKs Are Present in Regenerating Muscle Tissue and Show Temporal- and Age-Dependent Responses to Injury

Our study is the first to probe for endogenous CTKs in regenerating muscle tissue and identifies that CTKs are indeed found endogenously in rodent tissues, and that CTK levels fluctuate in response to injury and aging. Our data also suggests that of the four CTKs detected, 2-MeSiPR is responsive to injury and aging, as this CTK was significantly enriched in the injured muscle of 3-month-old mice, but no induction was seen in the older mice (Figure 1B). 2-MeSiPR is a particularly interesting CTK as in mammals, this form of modified CTK is present in mitochondria, thus a byproduct of mitochondrial tRNA degradation [23,24]. In mice, deletion of the enzyme responsible for 2-methylthio modifications, Cdk5rap1, impairs mitochondrial protein translation and leads to myopathy [24]. The timing of 2-MeSiPR induction in our injury model is therefore likely of biological significance, as this CTK returns to baseline levels by 7 dpi. The biphasic profile of 2-MeSiPR parallels the inflammatory response present in young mice wherein inflammation ramps up at 1–3 dpi, and resolves at approximately 7 dpi, but in older mice this reaction is delayed and weaker [19]. Like Cdk5rap1, mutations or deficiency in the enzyme responsible for adenosine prenylation, tRNA isopentenyltransferase 1 (TRIT1), is associated with mitochondrial dysfunction and the human disease combined oxidative phosphorylation deficiency 35 (COXPD35) [25,26,27,28,29]. The biological significance of endogenous BA and DZ9G in muscle is unknown. However, in plants 9G-CTKs are considered temporarily inactivated conjugates, and may reflect CTK synthesis rates, as they tend to accumulate gradually over time [30].

### 3.2. The Effect of Exogenous iP Types on Cell Growth, Viability, and Differentiation of C2C12 Myoblasts

The dose-dependent proliferative response of C2C12 cells to iP (the free base) is similar to a study conducted using rat L6 myoblasts which demonstrated that treatment with 5.0–10 µM of iP stimulated proliferation, but higher concentrations, such as 100 µM, did not [21]. On the other hand, we found that the riboside form (iPR) was more potent at inhibiting myogenesis and proliferation in cultured muscle cells than the free base (iP). This suggests a form-specific function between the free-base and riboside. As the iP types have been shown to act as AMP mimetics to suppress proliferation in epithelial cells [13,31], one possibility is that the riboside can be more efficiently converted to its nucleotide form, to iPMP, which then may function as an AMP mimetic. This conversion is relatively fast, as in endothelial cells, exogenous iPR is taken up and converted to iPMP by cells within 1 h, and activates AMPK by 6 h [13]. In contrast, Ogawa et al. recently showed through protein crystal simulation and in vitro AMPK kinase assays that iPMP inhibits AMPK activity [32]. Future studies in skeletal muscle should assess AMPK activity in response to exogenous iPR via AMPK phosphorylation and downstream effectors to clarify whether iPR modulates AMPK to repress proliferation and differentiation via conversion to iPMP or directly as a riboside.

As we previously observed that pro-inflammatory stress via lipopolysaccharide (LPS) increases extracellular isopentenyladenine (iP) types [18], and our current study demonstrates an increase in 2-MeSiPR in response to muscle injury, at least in young mice, one possibility is that iPR or its related types participate in paracrine signaling to control the proliferation and differentiation of neighboring cells in response to tissue damage. These possibilities suggest that the CTK biosynthesis network in mammals may be more complex than currently appreciated, and that exogenous applications of CTKs could have important roles in modulating skeletal muscle repair in vivo. For example, if iP can induce satellite cell proliferation in vivo, it could be used to promote muscle repair post-injury or in aging, where satellite cell number and function are reduced. On the other hand, improved myogenesis in vivo may be achieved by mitigating the inhibitory effects of iPR. Our data suggests that further analysis of the role of endogenous or exogenous iP and iPR on muscle repair in vivo could be of future therapeutic relevance.

### 3.3. The Impact of Exogenous Kinetin Types on Growth, Viability, and Differentiation of Myogenic C2C12

Kinetin riboside demonstrated notable dose-dependent cytotoxic effects, as indicated by a marked reduction in cell differentiation, proliferation and viability. Like the iP types, the effects of the riboside were generally stronger than the free base form of kinetin. This is in contrast with findings from Mielcarek et al. who reported that kinetin (the free base) could increase muscle cell differentiation [22]. The inhibitory effects of kinetin riboside on wound healing in the scratch and differentiation assays were striking and align with previous findings by Mielcarek et al. who reported similar cytotoxic effects of kinetin riboside, though their experimental approach was different from our C2C12 culture conditions [22]. It is also interesting that the effects of kinetin riboside were stronger than iPR, suggesting different mechanisms or potencies for these closely related molecules. The mechanisms behind these inhibitory effects are unclear and further complicated by the fact that kinetin is not common in biological systems and was not detected by our group in a previous study using culture myoblasts [18], nor in the present in vivo analysis (Figure 1). For both iP and kinetin types, it is also important to consider that the in vitro experiments may reflect supraphysiological concentrations and thus may not be representative of the in vivo, endogenous function of these molecules.

In summary, this study identifies a biological function for CTKs in muscle cell proliferation, differentiation, and injury responses, independent of the adenine/adenosine building block on which they arise. Instead, the side chain modification (isoprenyl or aromatic chain) and, whether it is a free base or riboside, strongly influence muscle cell proliferation.

## 4. Materials and Methods

### 4.1. Reagents

All adenine-based compounds, including adenine, adenosine, kinetin, kinetin riboside, N6-isopentenyladenine (iP), and isopentenyladenosine (iPR), were of analytical grade and were purchased from OlChemim Ltd. (Olomouc, Czech Republic), with the exception of adenosine (Millipore Sigma, Burlington, ON, Canada; A4036-5G). All reagents were solubilized in LC-MS Grade dimethyl sulfoxide (DMSO, 85190; Thermo Fisher Scientific, Mississauga, ON, Canada) and stored at −20 °C in the dark prior to use.

### 4.2. Muscle Injury

Animal procedures were approved by the Trent University Animal Care Committee. Male C57BL/6N mice aged 3- and 12 months (Charles River Laboritories, Sherbrooke, QC, Canada) received two 50 µL injections of 10 µM cardiotoxin (Latoxan/Cedarlane, Burlington, ON, Canada) into the gastrocnemius muscle. The contralateral control muscle was injected with equal volumes of Phosphate-Buffered Saline (PBS). Mice were randomized to be sacrificed either 3 or 7 days post-injury (*n* = 3 per timepoint, per age). The gastrocnemius muscle was isolated and snap frozen in liquid nitrogen. For mass spectrometry analysis, the muscle was then pulverized into a fine powder using a mortar and pestle over liquid nitrogen. The dry weight of each pulverized muscle tissue used for downstream mass spectrometry was recorded.

### 4.3. Cytokinin Extraction, Purification, and Quantification

Samples were extracted and purified for CTKs using 1.5 mL of ice-cold 50% acetonitrile (ACN; CH_3_CN: H_2_O, *v*/*v*) and hydrophilic–lipophilic balance (VIOLET™ HLB, 200mg/6 mL, 40 μm; Canadian Life Science, Peterborough, ON, Canada) solid-phase extraction cartridges. Prior to extraction, each sample was spiked with a panel of stable isotope-labeled CTK internal standards, as described previously [20]. Cartridges were conditioned sequentially with 5 mL methanol (MeOH; CH_3_OH), 5 mL ultrapure water (H_2_O; Millipore system, Etobicoke, ON, Canada), and 3 mL of 50% ACN. Sample supernatants were loaded onto the cartridges, and the flow-through was collected. Subsequently, 2 mL of 30% acetonitrile was applied, and the eluate was combined with the flow-through. The pooled fractions were dried under vacuum at ambient temperature using a speed-vac concentrator. Residues were reconstituted in 300 µL of 0.08% acetic acid in 5% ACN (CH_3_COOH:CH_3_CH: H_2_O, *v*/*v/v*) solution and centrifuged at 3000× *g* for 10 min. Supernatants were transferred into insert-equipped vials for analysis. CTKs were quantified by high-performance liquid chromatography coupled with high-resolution tandem mass spectrometry (HPLC-MS) using a Thermo Ultimate 3000 UHPLC system connected to a Q-Exactive™ Orbitrap mass spectrometer equipped with a heated electrospray ionization (HESI) source (Thermo Scientific, San Jose, CA, USA). A 25 µL aliquot was injected onto a reversed-phase C18 column (Kinetex 2.6 µm C18 100 Å, 2.1 × 50 mm; Phenomenex, Torrance, CA, USA). Separation was achieved with a binary solvent system: (A) water containing 0.08% acetic acid and (B) acetonitrile containing 0.08% acetic acid, at a flow rate of 0.5 mL/min. The gradient program was as follows: 5% B for 0.5 min, linear increase to 45% B over 4.5 min, ramp to 95% B over 0.1 min, hold at 95% B for 1 min.

Mass spectrometric detection was performed in positive ion mode using parallel reaction monitoring (PRM) at a resolution of 35,000 (full width at half-maximum, fwhm) at *m*/*z* 200. Source parameters were as follows: sheath gas, 30 arbitrary units; auxiliary gas, 8 arbitrary units; maximum spray current, 100 µA; auxiliary gas heater temperature, 450 °C; S-lens RF level, 60; spray voltage, 3.9 kV. PRM settings included an AGC target of 3 × 10^6^, maximum injection time of 128 ms, isolation window of *m*/*z* 1.2, and normalized collision energies individually optimized for each analyte [20]. Data were processed using Thermo Xcalibur v3.0.63 (Thermo Scientific, San Jose, CA, USA). Quantification was performed by isotope dilution analysis, based on recovery of deuterium-labeled internal standards, with peak areas used for relative and absolute determination of CTK levels.

### 4.4. Cell Culture

Murine C2C12 myoblasts (ATCC) were cultured in Dulbecco’s Modified Eagle’s Medium (DMEM; D6422, Millipore Sigma, Burlington, ON, Canada) supplemented with 10% fetal bovine serum (FBS; Gibco, 16000044, Thermo Fisher Scientific, Mississauga, ON, Canada) and 1% penicillin-streptomycin (P/S; 15140122, Thermo Fisher Scientific, Mississauga, ON, Canada) at 37 °C in a humidified 5% CO_2_ incubator.

### 4.5. C2C12 Myoblast Proliferation

Cells were seeded in 96-well plates at two densities (~1 × 10^4^ or ~0.6 × 10^4^ cells/well) and allowed to adhere for 24 h. After reaching 90–95% and 60–65% confluence, for differentiation or prolfieration assays, the cells were treated with iP, iPR, kinetin, KR, adenine, and adenosine at 0.1, 1.0, and 10 µM concentrations in serum-free GM for 24 h. The final DMSO concentration was maintained at ≤0.1% in all experiments.

### 4.6. C2C12 Myoblast Differentiation

For differentiation, C2C12 myoblasts were cultured in 96-well plates and maintained in GM for 24 h. Upon reaching 90–95% confluence, GM was replaced with differentiation medium (DM) comprised of sodium pyruvate-deficient DMEM (Gibco, 11965092, Thermo Fisher Scientific, Mississauga, ON, Canada) supplemented with 2% horse serum (Cytiva Hyclone, SH3007403, Fisher Scientific, Whitby, ON, Canada) and 1% P/S. The cells were subsequently exposed to the candidate compounds for 144 h, with fresh medium containing candidate treatments replenished daily.

### 4.7. Measurements of Cell Proliferation

DNA synthesis and proliferation were assessed using a 5-bromo-2′-deoxyuridine (BrdU) incorporation assay (Cell Signaling Technology, Whitby, ON, Canada; #6813S) following the manufacturer’s protocol. Briefly, C2C12 cells were treated with compounds in serum-free GM for 24 h, followed by BrdU addition and a 4 h incubation at 37 °C. Absorbance was measured at 450 nm using a BioTek Synergy™ HTX Multi-Mode Microplate Reader (Gen5 v.3.12.08).

### 4.8. Measurements of Cell Viability

Cell viability was assessed using a colorimetric Cell Counting Kit-8 (CCK-8; ab228554, Abcam, Waltham, MA, USA). Cells at ~90–95% and ~60–65% confluence were exposed to the candidate compounds in either serum-free or normal GM for 24 h. Following treatment, CCK-8/WST-8 reagent was added and incubated for 3 h, after which the absorbance was measured at 460 nm. Readings from blank wells (cell-free wells) were subtracted from absorbance mesreuments and we represent these as “Corrected O.D.” where O.D.represents optical density.

### 4.9. Scratch Wound Assay

A confluent monolayer of C2C12 cells, with approximately 90–95% coverage, was subjected to a scratch assay using a sterile 10 µL pipette tip. Following the removal of detached cells, fresh GM containing 10 μM of each treatment was applied. The progression of wound closure was monitored at 0-, 12-, and 24 h post-scratch utilizing a Nikon ECLIPSE Ti2 inverted microscope. Subsequent image analysis was conducted using ImageJ software. Using ImageJ (https://imagej.net/ij/ accessed on 13 October 2025), the initial scratch width at 0 h served as the baseline for wound healing analysis. Distances at 12 and 24 h were normalized to the 0 h distance for each well.

### 4.10. Immunofluorescence and Myogenic Indices Determination

C2C12 myoblasts were seeded in 24-well plates, differentiated for 144 h with candidate compounds, and fixed in 100% ice-cold methanol for 15 min at 4 °C. Cells were blocked with 0.5% bovine serum albumin (BSA) in D-PBS, then incubated overnight with anti-myosin heavy chain (MyHC) primary antibody (1:20 dilution, a gift from John McDermott, York University) followed by Alexa Fluor^®^ 488 secondary antibody (4416S, 1:2000 dilution from New England BioLabs, Whitby, ON, Canada). Nuclei were stained with DAPI (1 µg/mL). Fluorescent images were captured using a Nikon ECLIPSE Ti2 microscope and analyzed using the ImageJ software. The differentiation and fusion indices were calculated based on MyHC expression. The differentiation index was defined as the percentage of nuclei in myotubes expressing myosin heavy chain divided by the total number of nuclei in the field of view. The fusion index was calculated as the percentage of nuclei residing in a cell that expressed myosin heavy chain and contained two or more nuclei divided by the total number of nuclei in the cells [19].

### 4.11. Statistical Analyses

Statistical analyses were performed using GraphPad Prism 9.5.1 with one-way ANOVA followed by Dunnett’s post hoc test (*p* < 0.05) for *in vitro* analyses, and a two-way ANOVA followed by Sidak’s post hoc test (*p* < 0.05) for *in vivo* cytokinin analysis. Results are presented as mean ± standard deviation (SD).

## Data Availability

The original contributions presented in this study are included in the article. Further inquiries can be directed to the corresponding author(s).
